# Autoreactive IgE Is Prevalent in Systemic Lupus Erythematosus and Is Associated with Increased Disease Activity and Nephritis

**DOI:** 10.1371/journal.pone.0090424

**Published:** 2014-02-28

**Authors:** Barbara Dema, Christophe Pellefigues, Sarfaraz Hasni, Nathalie Gault, Chao Jiang, Tiffany K. Ricks, Michael M. Bonelli, Jörg Scheffel, Karim Sacré, Mathieu Jablonski, Delphine Gobert, Thomas Papo, Eric Daugas, Gabor Illei, Nicolas Charles, Juan Rivera

**Affiliations:** 1 Laboratory of Molecular Immunogenetics, National Institute of Arthritis and Musculoskeletal and Skin Diseases, National Institutes of Health, Bethesda, Maryland, United States of America; 2 Institut National de la Santé et de la Recherche Médicale U699, Université Paris Diderot, Paris, France; 3 Office of the Clinical Director, National Institute of Arthritis and Musculoskeletal and Skin Diseases, National Institutes of Health, Bethesda, Maryland, United States of America; 4 Department of Internal Medicine, Hôpital Bichat, Assistance Publique-Hôpitaux de Paris, Université Paris Diderot, Faculté de Médecine site Bichat, Paris, France; 5 Department of Nephrology, Hôpital Bichat, Assistance Publique-Hôpitaux de Paris, Université Paris Diderot, Faculté de Médecine site Bichat, Paris, France; 6 Clinical Research Unit, Hôpital Bichat, Assistance Publique-Hôpitaux de Paris, Université Paris Diderot, Faculté de Médecine site Bichat, Paris, France; 7 Sjogren’s Syndrome Clinic, National Institute of Dental and Craniofacial Research, National Institutes of Health, Bethesda, Maryland, United States of America; 8 Lymphocyte Biology Section, National Institute of Arthritis and Musculoskeletal and Skin Diseases, National Institutes of Health, Bethesda, Maryland, United States of America; INSERM-Université Paris-Sud, France

## Abstract

The presence of autoantibodies in systemic lupus erythematosus, particularly those of the IgG subclass, have long been associated with disease onset and activity. Here we explored the prevalence of autoreactive IgE in SLE and its relevance to disease in French (n = 79) and United States (US) (n = 117) cohorts with a mean age of 41.5±12.7 and 43.6±15.3 years and disease duration of 13.5±8.5 and 16.6±11.9 years, respectively. Our findings show that approximately 65% of all SLE subjects studied produced IgE antibodies to the seven autoantigens tested. This positivity was increased to almost 83% when only those subjects with active disease were considered. SLE subjects who were positive for anti-dsDNA, -Sm, and -SSB/La -specific IgE showed a highly significant association in the levels of these antibodies with disease activity similar to that of the corresponding IgG's. A strong association of IgE autoantibodies with active nephritis was also found in the combined cohort analysis. A test of the predictive value of autoreactive IgE’s and IgGs for disease activity (SLE Disease Activity Index (SLEDAI) ≥4) revealed that the best predictors were dsDNA-specific IgE and IgG, and that the age of an SLE subject influenced this predictive model. The finding argue that the overall levels of IgE autoantibodies, independently or in combination with IgG autoantibodies, may serve as indicators of active disease.

## Introduction

Systemic Lupus Erythematosus (SLE) is a chronic systemic autoimmune disease characterized by the loss of immune tolerance to self-antigens, dysregulated autoantibody production, and multiple clinical manifestations [1]. A hallmark of the disease is the overproduction of antibodies to nuclear antigens (ANA), dsDNA, Sm, RNP, Ro (SSA), La (SSB), and some phospholipids [2]. The detection of ANA, anti-Ro, anti-La and anti-phospholipid antibodies can occur years in advance of the clinical manifestation and diagnosis of SLE, and these antibodies accumulate with time. In contrast, the presence of anti-dsDNA and anti-Sm antibodies usually precedes by a few months the clinical manifestations of SLE and thus its diagnosis [3]. While the appearance of the different autoantibodies has been associated with diverse clinical manifestations [4], the pathogenesis of disease is most closely linked to the presence of dsDNA-specific IgG antibodies [5] and kidney biopsies reveal that a large proportion of SLE patients, that develop glomerular nephritis, have kidney deposits of dsDNA IgG antibodies [6]. However, not all patients produce dsDNA IgG antibodies and not all of these autoantibodies are pathogenic even though their presence is one criterion for SLE diagnosis [7], thus what other isotypes of autoantibodies might be linked to disease activity and kidney pathology is still a topic of considerable interest.

IgE is the rarest of immunoglobulins in the blood with serum levels in a healthy human at approximately 150 ng/ml whereas that of IgG is 10 mg/ml. Normally, IgE functions to protect against various parasitic infections [8], but it is best known to mediate type I hypersensitivity since it binds the high affinity receptor for IgE (FcεRI) on mast cells and basophils (key cells in allergy) and upon allergen recognition causes the activation of these cells and the release of allergic mediators [9]. The presence of IgE autoantibodies has been described in some autoimmune diseases more than three decades ago [10], albeit there has been little advance towards understanding the role of IgE in autoimmunity. Generally, measurement of autoreactive IgE in diseases like SLE has been performed on small patient cohorts using methodologies with poor sensitivity for IgE detection, potentially providing a false perception of the prevalence of these autoantibodies. While some studies suggest that autoreactive IgE’s may contribute to autoimmune diseases like SLE and to the underlying pathologies [11], [12], the general importance of IgE autoantibodies in SLE has also been questioned [13] based on studies where a small proportion of SLE subjects showed detectable levels of autoreactive IgE’s [11]. Nonetheless, the prevalence of autoreactive IgE in SLE and its importance to disease activity and pathologies is underappreciated.

Recently, we reported that the presence of autoreactive (dsDNA) IgE is linked to activation of basophils, which function to amplify autoantibody production in SLE through support of plasma cell survival [12]. In a mouse model of spontaneous lupus-like disease (Lyn-deficient mice), we found that the presence of IgE in circulating immune complexes caused the activation of circulating blood basophils which, upon activation, home to the secondary lymphoid organs by expressing CD62L in their cell surface. In the lymph nodes and the spleen, basophils secreted cytokines (IL-4 and IL-6) and expressed MHC-II and BAFF, enhancing plasma cell survival and autoantibody production [12], [14]. Crossing of Lyn-deficient mice to IL-4-deficient or IgE-deficient mice resulted in a marked reduction in Ig autoantibody production and a failure of these double-deficient mice to develop the lupus-like phenotype. Thus, the findings argue for a functional role of autoreactive IgE in amplifying loss of self-tolerance through basophil-mediated support of plasma cell autoantibody production. Analysis of human disease also demonstrated the presence of dsDNA-specific IgE antibodies and activated basophils in a small cohort of SLE subjects [12]. dsDNA-specific IgE autoantibodies were associated with active disease and with lupus nephritis, suggesting that the observed role for autoreactive IgE and basophils in amplifying autoantibody production, in mouse models, might be a plausible immunological mechanism in the progression of human disease.

To further advance the understanding of IgE in SLE we measured the levels of IgE’s with specificity to the most common SLE antigens, dsDNA, Sm, SSA (Ro), and SSB (La) in a sample of 196 SLE subjects in France or the US with a mean age of 41.5±12.7 and 43.6±15.3 years and disease duration of 13.5±8.5 and 16.6±11.9 years, respectively (Table 1). The healthy controls used in this study had a mean age 33.1±8.8 and 47.3±12.7 in France and the US, respectively (Table 1). The findings show that autoreactive IgE’s are highly prevalent in SLE and that the presence of high levels of dsDNA-specific IgE are tightly linked to disease activity and to parameters of abnormal kidney function similar to dsDNA-specific IgG. We also uncovered autoreactive IgE’s, but not IgG’s, to novel self-antigens that are linked with hypocomplementemia, a serological marker of disease activity. The findings demonstrate that autoreactive IgE is prevalent in SLE and suggest that its detection together with anti-dsDNA IgG, may serve to better predict increased disease activity and/or poor kidney function.

**Table 1:** Characteristics and demographics of SLE patients and healthy controls in US and French cohorts.

	US		France	
Characteristic	SLE	Controls	SLE	Controls
	(n = 117)	(n = 79)	(n = 79)	(n = 32)
**Age (years) (Mean, SD)**	43.6±15.3	47.3±12.7	41.5±12.7	33.1±8.8
**Gender, Females n (%)**	99 (84.6)	30 (38.0)	70 (88.6)	15 (46.8)
**Ethnicity, n (%)**				
** European descent**	40 (34.2)	45 (56.9)	52 (65.8)	28 (87.5)
** African-American/African**	35 (29.9)	28 (35.4)	20 (25.3)	1 (3.1)
** Hispanic**	23 (19.6)	5 (6.3)	3 (3.8)	1 (3.1)
** Asian**	14 (11.9)	1 (1.3)	4 (5.1)	0
** Arabs**	1 (0.8)	0	0	0
** Unknown**	4 (3.4)	0	0	– (6.2)
**Disease duration (years), (mean, SD)**	16.6±11.9	–	13.5±8.5	–
**SLEDAI, IQR (25%, 75%)**	4 (0,4)	–	9 (0, 9)	-
**Current Prednisone dose (mg/day)**				
**IQR (25%, 75%)**	9.5 (0.5,10)	–	12.25 (1.25, 13.5)	–
**Missing values**	–		2	
**Immunosuppressive therapy, n (%)**				
** Hydroxychloroquine**	89 (76.1)	–	71 (91.0)	–
** Mycophenolate mofetil**	10 (8.5)	–	25 (33.8)	–
** Cyclophosphamide**	4 (3.4)	–	45 (60.8)	–
** Azathioprine**	22 (18.8)	–	11 (14.5)	–
** Cyclosporine**	2 (1.7)	–	0	–

IQR, Interquartile range.

## Materials and Methods

### Studied Cohorts

This collaborative study was approved by the Institutional Review Board of NIAMS (NIH, Bethesda, USA) and by the Comité Régional de Protection des Personnes (CRPP, Paris, France). A written consent was obtained from all healthy donors and SLE patients. In the US, samples were obtained from SLE patients enrolled in a long-term natural history study of SLE. In France SLE samples were obtained from in- and out- patients enrolled in a long term prospective study of SLE, and clinical data were used after approval by the Comission Nationale de l’Informatique et des Libertés (CNIL). All patients fulfilled the American College of Rheumatology classification criteria for SLE [7], [15]. Samples from “healthy” volunteers were obtained from the blood bank at the NIH Clinical Center and from the Université Paris Diderot, Faculté de Médecine site Bichat. The demographics and characteristics of all SLE patients and healthy controls are summarized in Table 1. The data included herein is from one sample provided by each individual SLE subject.

### Sample Collection

Whole blood from SLE and healthy donors was collected in 4 ml SST (serum-separating tube) tube for serum. Serum samples were aliquoted and kept at −80°C.

### Enzyme-linked Immunosorbent Assays (ELISAs)

The level of antibodies against the four common nuclear antigens (dsDNA, Sm, Ro/SSA, La/SSB) was measured by an ELISA. Horseradish peroxidase-conjugated antibodies to human IgE (Mouse monoclonal anti-human IgE, Immunology Consultants Laboratory) or IgG (Donkey anti-human IgG, Fc fragment specific, Jackson Immunoresearch Laboratory) were used for colorimetric detection was with tetramethylbenzidine substrate (TMB, Invitrogen) and optical density (OD) was measured at 450 nm. Positive values were considered as greater than two standard deviations over the mean absorption units obtained from samples of healthy individuals. dsDNA-specific IgG levels were quantified using standard laboratory procedures by the NIH Clinical Center and by the INSERM U699 Total levels of IgE were determined by ELISA (Calbiotech, for US; Bethyl Laboratories for France) following the manufacturers’ instructions.

To determine the level of IgG and IgE autoantibodies to CLIP4, MPG and APEX nuclease 1, we used purified recombinant human proteins, CLIP4 (RSNL2 protein, Abnova), MPG (DNA-3 methyladenine glycosylase, Mybiosource), and APEX nuclease 1 (DNA apurinic or apyrimidinic site lyase, Mybiosource) in ELISA. Detection of reactive autoantibodies was as described above.

### Protein Microarray

A Protoarray Human Protein Microarray v5.0 (Invitrogen Life Technologies) with over 9000 purified human proteins was used for novel antigen discovery. The purified recombinant proteins spotted on nitrocellulose-coated glass slides were reacted with the serum of SLE subjects or healthy controls and subsequently incubated with biotinylated goat anti-human IgE antibody (5 µg/ml) (Vector Laboratories) to detect IgE antibody binding. Then either an AlexaFluor 647-conjugated streptavidin (Invitrogen, 1 µg/ml) or an AlexaFluor 647-conjugated anti-V5 antibody (Invitrogen, 0.1 µg/ml) was added for detection and normalization, respectively. Arrays were scanned using fluorescent microarray GenePix 4000B Scanner and GenePix 6.0 software was used for data acquisition. Data analysis was done using Invitrogens proprietary Protoarray Prospector software. For each protein spotted in every array, Z-Score, Z-factor, CI-P value (Chebyshevs Inequality p-Value) was measured. M-Statistic is used to identify those proteins which show a significant differential signal between two populations. The “cutoff” value used corresponded to the value of 200 RFU above the M-Statistic signal threshold established for a specific protein.

### Statistics

Student *t* test or a non-parametric Mann-Whitney U-test (when necessary) was used to compare SLE and control samples. A Chi2 assessment of the non-parametric Fisher exact test was used to determine the association of autoreactive IgE with active nephritis as well as the association of autoreactive IgE with active disease. One-way ANOVA with Bonferroni correction test or Kruskal-Wallis with Dunns correction test was applied when more than two groups were compared. Mean and SEM is shown in the scatter plot graphs. The analysis was performed using Graphpad Prism 5.0. Grubbs’ outlier test was used to assess the outliers. In some instances some outliers detected in the healthy control group were excluded from the analysis because of the identification of allergic disease or a urine infection and no clinical indications of these conditions in the studied SLE subjects. For forest plot analysis, a fixed effects model was applied and Hedges g statistics was used to calculate the standardized mean difference (SMD).

Stepwise Logistic Regression offered by IBM SPSS Statistics 20 software was used to determine a predictive model of disease activity (Not active, SLEDAI<4 vs Active, SLEDAI≥4). Probability to enter a predictor in the model p<0.1 and to remove it p>0.05. Model 1 included as predictors: positive levels of dsDNA IgE, Sm IgE, SSA/Ro IgE, SSB/La IgE, dsDNA IgG, Sm IgG, SSA/Ro IgG and SSB/La IgG; as confounders age, gender and ethnicity (African American/African, European descendent, Hispanic and others). Model 2 included as predictors: positive levels of autoreactive IgEs, autoreactive IgGs (positive for at least one of the autoantibodies against the four common antigens); as confounders age, gender and ethnicity. Factors not affecting the models were eliminated from consideration. ROC Curve with AUC (area under the curve) analysis and Youden’s J Index was calculated by IBM SPSS in order to determine the optimal predictive model selection.

## Results

### IgE Auto-antibodies to Common SLE Antigens are Prevalent

The characteristics and demographics of both French and US SLE subjects studied herein are described in Table 1. French SLE subjects were recruited through a long term prospective study of SLE from a primary care center whereas US SLE subjects were part of a long term natural history study of SLE in a secondary care center. Thus, while the mean age of each cohort was similar, the mean years of disease duration was somewhat less in the French subjects (13.5±8.5) versus US subjects (16.6±11.9) and French subjects had a higher SLEDAI score (>9) than US subjects (>4). As might be expected, given that the US subjects were recruited from a secondary care center, only 13 of the 117 US subjects (∼11%) had active nephritis whereas 30 of the 79 French subjects (∼38%) were active. Treatment regimens were similar in the two cohorts with the exception that a greater number of French subjects (45) were on cyclophosphamide than US subjects (4). The two cohorts also differed in their ethnic composition with fewer Hispanics and Asians present among the French subjects.

**Figure 1:**
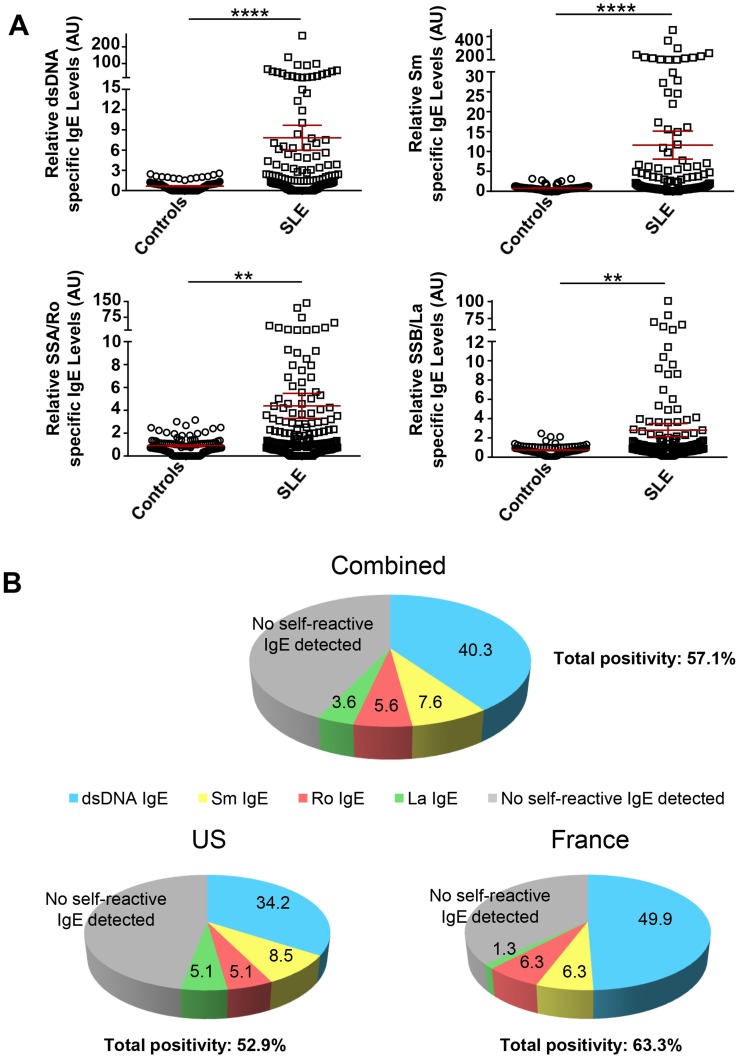
Elevated levels of dsDNA-, Sm-, SSA/Ro- and SSB/La-specific IgE antibodies in SLE subjects versus healthy controls. **A.** Relative levels from a combined analysis of the US and French SLE cohorts for IgE antibody levels to the four aforementioned antigens. Mann Whitney test was used to compare the different groups. Mean ± SEM is shown. AU, Arbitrary Units. **p<0.01; ****p<0.0001. **B.** Prevalence of IgE autoantibodies to the aforementioned four common SLE autoantigens. Percentage of patients positive for IgE autoantibodies (2SD over the mean of healthy controls) to at least one of the four autoantigens tested. dsDNA-specific IgE (as the most prevalent autoreactive IgE) was used as the reference. The percent of anti-Sm IgE positive individuals are those without dsDNA-IgE, percent of anti-SSA/Ro positive individuals are without dsDNA- and Sm-IgE’s, and anti-SSB/La positive individuals are those without dsDNA, Sm and SSA/Ro reactivties. Therefore, patients in the group of those positive for anti-dsDNA IgE were also positive for the rest of IgE autoantibodies tested, but none of the subjects in the anti-SSB/La IgE positive group had detectable IgE autoantibodies for dsDNA, Sm, or SSA/Ro.

It was not known if IgE autoantibodies are generated to all of the antigens most frequently detected in the serologic profile of SLE. Thus, we assayed for the presence of dsDNA, Sm, SSA/Ro and SSB/La in both US and French SLE cohorts. IgE autoantibodies to all four self-antigens were present with anti-dsDNA and anti-Sm IgE antibodies having the most significant association with SLE relative to healthy controls (Figure 1A). Analysis of the overall frequency of all SLE patients with autoreactive IgE positivity (a minimum of one auto-antigen specific IgE) demonstrated a frequency of over 57% (Figure 1B) with the US SLE cohort approaching 53% and the French cohort at 63%. Thus, 47% of the US cohort and 37% of the French cohort did not show IgE reactivity to one of the four auto-antigens tested. Of the four autoantigen specificities, dsDNA specific-IgE was the most frequent with 34.2% and 49.4% of the US and French cohorts, respectively, having this autoantibody (Figure 1B). To test whether the two cohorts had similar characteristics with regards to autoreactive IgE’s we performed a forest plot (Figure S1 in File S1) and found no heterogeneity (I^2^ = 0%) in the levels of IgE autoantibodies among the cohorts and no significant difference was observed in the effect of the autoreactive IgE on the two cohorts relative to healthy controls as shown by the overall standardized mean difference (SMD) for each individual autoreactive IgE. This argues that the two cohorts showed similar differences in levels of autoreactive IgE relative to healthy controls and that the distribution of the detected autoreactive IgE is similar in these two cohorts. No meaningful differences in the levels of IgE autoantibodies were detected when either gender or ethnicity were considered and no correlation was found between the total levels of IgE and the levels of IgE autoantibodies in these cohorts (data not shown).

### IgE Autoantibodies are Associated with Increased Disease Activity

**Figure 2:**
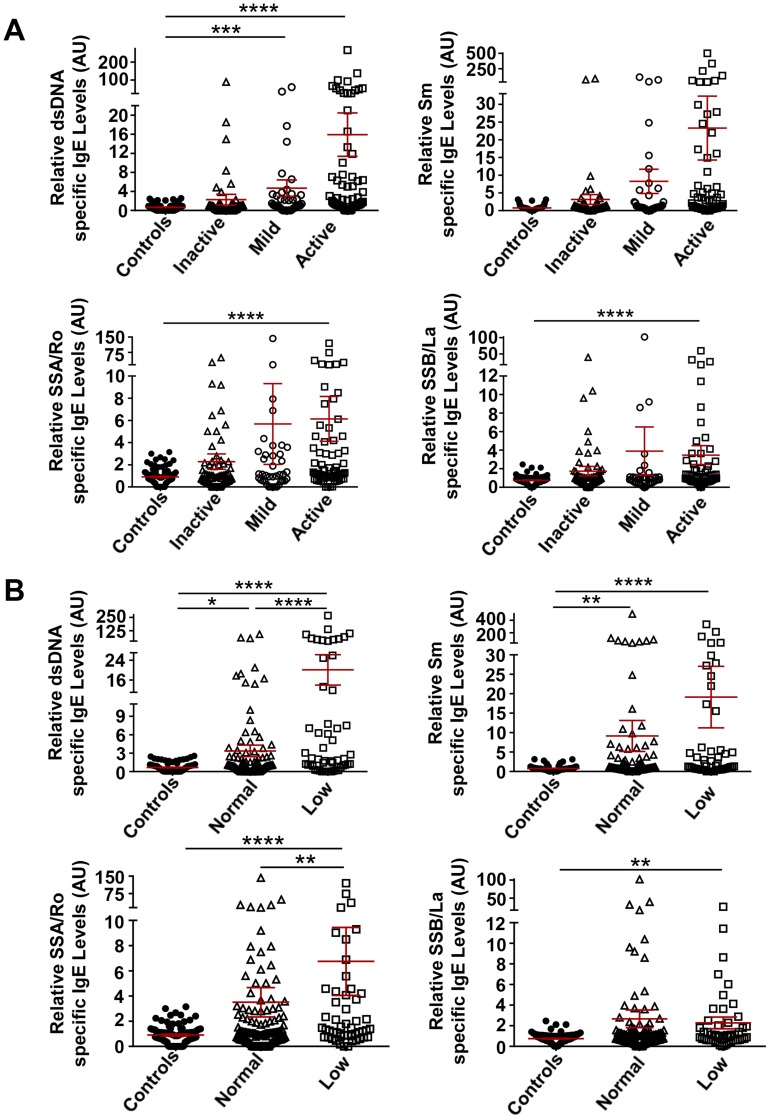
Comparison of the levels of dsDNA-, Sm-, SSA/Ro- and SSB/La-specific IgEs with disease activity. **A.** Combined analysis of the levels of autoreactive IgE’s in the US and French SLE cohorts with disease activity as defined by SLEDAI score. Inactive, SLEDAI  = 0; Mild, SLEDAI >0 to <4; Active, SLEDAI ≥4. **B.** Combined US and French SLE cohort analysis of IgE autoantibodies with complement levels in serum. Low levels of complement were considered as C3<80 mg/dl or C4<15 mg/dl. Kruskal-Wallis with Dunn’s multiple comparisons test was used to compare the different groups. Mean ± SEM is shown. AU, Arbitrary Units. *p<0.05; **p<0.01; ***p<0.001; ****p<0.0001.

The relationship between disease activity and the presence of any one of the four auto-antigen specific IgE’s was explored. Disease activity was based on a SLEDAI score of ≥4 as active disease, >0 to <4 for mild disease, and 0 for inactive. As shown in Figure 2A, SLE subjects with autoreactive IgE’s were most frequently found in the active disease group. SLE subjects with mild disease also had significantly elevated levels of IgE autoantibodies to dsDNA (Figure 2A).

To further verify the association of autoreactive IgE’s with disease activity, we analyzed its relationship to hypocomplementemia, a commonly used serological marker of increased disease activity or disease relapses [16], [17]. The levels of IgE autoantibodies to the tested autoantigens (dsDNA, Sm, SSA/Ro, SSB/La) were significantly elevated in SLE subjects with hypocomplementemia (C3<80 mg/dl, C4<15 mg/dl) (Figure 2B). However, dsDNA and Sm-specific IgE’s were also significantly elevated in SLE subjects with normal complement levels. When the cohorts were analyzed individually, only ds-DNA-specific IgE showed a significant association with hypocomplementemia in both the French and US cohorts (data not shown), demonstrating that this autoreactive IgE is most significantly associated with hypocomplementemia.

While more than 50% of SLE subjects had autoreactive IgE to one or more of the four common SLE auto-antigens, the prevalence of these IgE’s in active SLE subjects (SLEDAI ≥4) was 73.7% and 74.1% for the French and US subjects, respectively, with dsDNA specific-IgE being the most prevalent (US = 62.8%, French = 63.2%) (Figure S2A in File S1). When we analyzed the prevalence of these autoreactive IgE’s in SLE subjects with hypocomplementemia, it was higher in the French cohort (90%) than in the US cohort (72%) with dsDNA-specific IgE being found in 60% of the US and 80% of the French SLE subjects with hypocomplementemia (Figure S2B in File S1). These findings argue that the presence of autoreactive IgE’s (and in particular dsDNA-specific IgE) in SLE may be a reasonable clinical indicator of increased disease activity.

### IgE Autoantibodies are Associated with Active Nephritis and Other Clinical Parameters of Disease Activity

**Figure 3:**
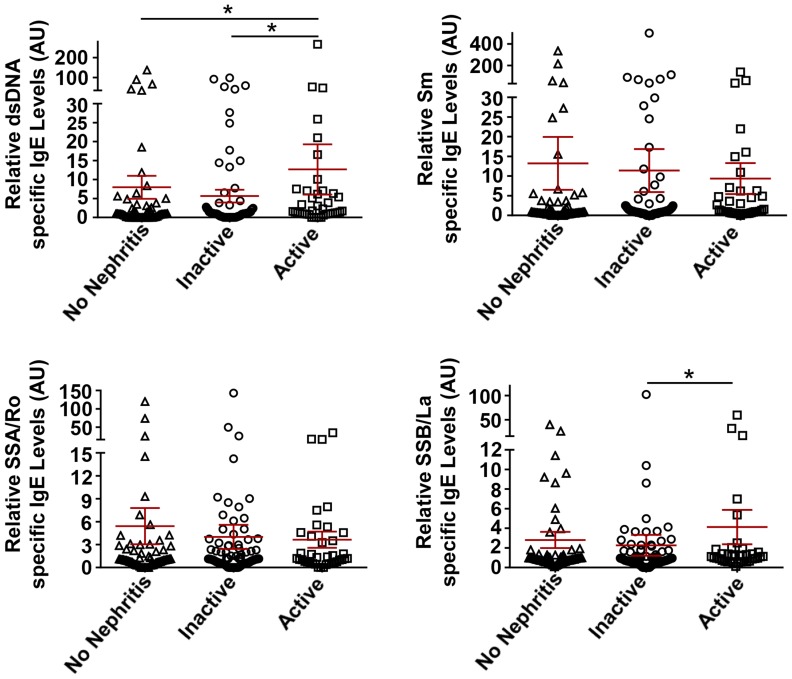
Association of autoreactive IgE’s with lupus nephritis. Relative levels of dsDNA-, Sm-, SSA/Ro- and SSB/La-specific IgE autoantibodies in the combined US and French SLE cohort with no nephritis, inactive or active nephritis. Kruskal-Wallis with Dunn’s multiple comparisons test was used to compare the different groups. Mean ± SEM is shown. AU, Arbitrary Units. *p<0.05.

Forty three of the 196 SLE subjects under study were determined to have active nephritis based on a protein/creatinine ratio of ≥0.5, positivity for dsDNA IgG, and hypocomplementemia. As shown in Figure 3, a significant association of active nephritis with increased levels of dsDNA–specific IgE’s (no nephritis vs active nephritis, p = 0.02) was observed. If the analysis is limited to those individuals in the combined cohort who were positive for increased levels of autoreactive IgE’s (Table S1 in File S1), the association with dsDNA-IgE was highly significant. Univariate analysis of the individual cohorts (Table S1 in File S1) revealed no significant association in the US SLE subjects, even for dsDNA-specific IgE (p = 0.20). In contrast, in French cohort where 30 of 79 SLE subjects (∼38%) had active nephritis, both dsDNA and Sm- specific IgE’s showed a significant association (p = 0.017 and 0.021, respectively) with this clinical diagnosis (Table S1 in File S1).

Since the titer of circulating IgG autoantibodies (in particular dsDNA-specific IgG) is known to be associated with SLE disease activity, we set out to compare the relationship of autoreactive IgG and IgE with disease activity. Analysis of SLE subjects, with active disease, having positivity for autoreactive IgE or IgG to the four common SLE autoantigens showed a similar significance (p<0.0001) in the association of anti-dsDNA IgE or IgG with active disease (Table 2). However, anti-Sm IgE showed a more significant association with active disease than anti-Sm IgG (p = 0.0008 vs. p = 0.013) and anti-SSB/La IgE also showed a significant (p = 0.0044) association whereas the association of its IgG counterpart did not achieve significance (Table 2).

**Table 2:** Correlation of positive levels of autoreactive IgEs and IgGs with disease activity (Active SLEDAI≥4, No Active SLEDAI<4). Analysis of the combined US and French cohorts.

		Anti-dsDNA IgE		Anti-Sm IgE		Anti-Ro/SSA IgE		Anti-La/SSB IgE		Autoreactive IgEs	
		Neg	Pos	Neg	Pos	Neg	Pos	Neg	Pos	Neg	Pos
**Active Disease**	**No**	90	33	93	28	92	29	99	22	51	32
		(73.2%)	(26.8%)	(76.8%)	(23.1%)	(76.1%)	(23.9%)	(81.8%)	(18.2%)	(61.4%)	(38.5%)
	**Yes**	27	46	38	33	42	29	45	26	19	54
		(36.9%)	(63.1%)	(53.5%)	(46.5%)	(59.2%)	(40.8%)	(63.4%)	(36.6%)	(26.0%)	(73.9%)
	**p value**	p<0.0001		p = 0.0008		p = 0.014		p = 0.0044		p<0.0001	
	**OR (95% CI)**	4.64 (2.49–8.64)		2.84 (1.54–5.41)		2.19 (1.16–4.12)		2.6 (1.33–5.07)		4.53 (2.28–8.98)	
		**Anti-dsDNA IgG**		**Anti-Sm IgG**		**Anti-Ro/SSA IgG**		**Anti-La/SSB IgG**		**Autoreactive IgGs**	
		**Neg**	**Pos**	**Neg**	**Pos**	**Neg**	**Pos**	**Neg**	**Pos**	**Neg**	**Pos**
**Active Disease**	**No**	85	38	75	46	72	49	95	26	38	45
		(69.1%)	(31.2%)	(61.9%)	(38.0%)	(59.5%)	(40.5%)	(78.5%)	(21.5%)	(45.8%)	(54.2%)
	**Yes**	25	48	31	40	29	42	48	23	10	63
		(34.2%)	(65.7%)	(43.7%)	(56.3%)	(40.8%)	(59.1%)	(67.6%)	(32.3%)	(13.7%)	(86.3%)
	**p value**	p<0.0001		p = 0.013		p = 0.012		p = 0.09		p<0.0001	
	**OR (95% CI)**	4.29 (2.32–7.95)		2.10 (1.16–3.82)		2.13 (1.17–3.86)		1.75 (0.90–3.38)		5.32 (2.40–11.78)	

OR, Odds Ratio.

CI, Confidence Interval.

Analysis of the predictive value of autoreactive IgE’s or IgG’s for active disease in SLE subjects demonstrated that anti-dsDNA IgE and anti-dsDNA IgG were significant in predicting SLE subjects with active disease (Table 3, AUC = 0.769, Youden’s J = 0.37), compared to the individual analysis of each of the predictors (dsDNA-IgE: AUC = 0.72, Youden’s J = 0.36; dsDNA-IgG: AUC = 0.737, Youden’s J = 0.347). However, anti-dsDNA IgG was a modestly stronger predictor (OR adjusted = 3.43 versus OR 2.77 for anti-dsDNA IgE) perhaps due to inclusion of dsDNA-IgG’s as a determinant of SLEDAI. Inclusion of autoreactive IgG’s and IgE’s with other specificities (Sm, SSA/Ro, SSB/La) reduced the overall sensitivity of the model indicating that these were not strong indicators of disease activity (data not shown). Introduction of ethnicity, gender, and age as confounding factors revealed that only age influenced the predictive model (Table 3). Thus, the findings show that, like anti-dsDNA IgG, anti-dsDNA IgE is useful in predicting disease activity and the use of both of these autoantibodies increases the predictive ability (Table 3).

**Table 3:** Adjusted results of Multivariate analysis by Stepwise Logistic Regression.

		p value	OR	95% CI	AUC	Youdeńs J
**Model 1**	dsDNA IgE	p = 0.004	2.77	1.38–5.55		
	dsDNA IgG	p<0.001	3.43	1.72–6.85	0.769	0.37
	Age	p = 0.008	0.97	0.94–0.99		
**Model 2**	IgEs Pos	p = 0.011	2.39	1.22–4.67		
	IgGs Pos	p = 0.022	2.58	1.14–5.81	0.735	0.32
	Age	p = 0.003	0.97	0.94–0.99		

ROC curve and Youden’s J Index analysis of both models.

OR, Odds Ratio.

CI, Confidence Interval.

AUC, Area under the curve.

### Discovery of Novel Auto-antigens for IgE in SLE and their Relationship with Disease Activity

**Figure 4:**
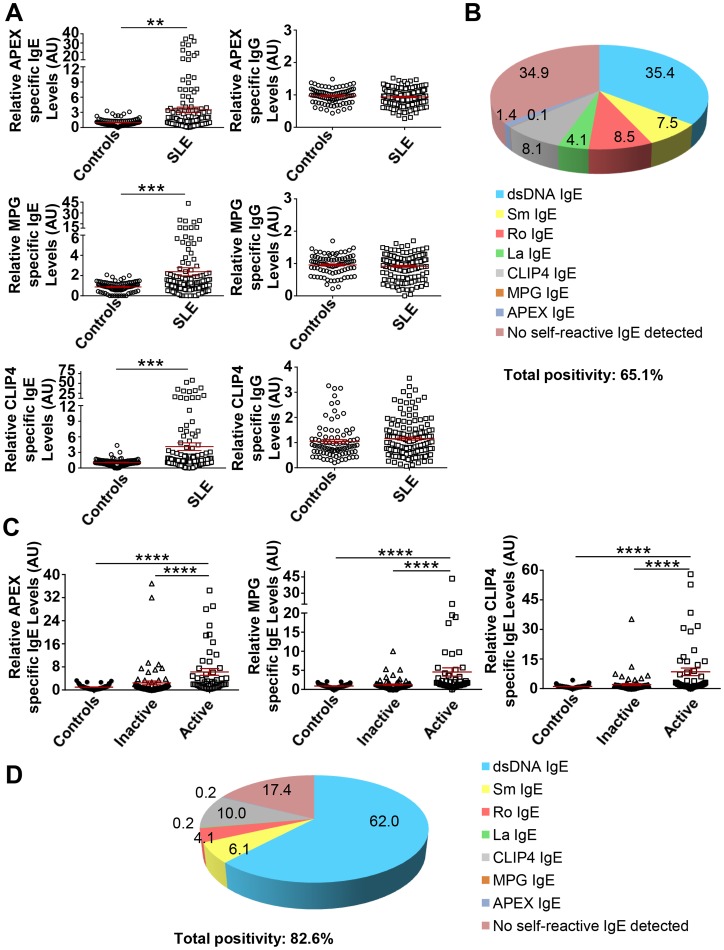
Identification of novel IgE autoantigens in SLE, overall prevalence, and association of all tested autoreactive IgE’s with SLE disease activity. **A.** Relative levels of APEX, MPG and CLIP4 specific IgEs and IgGs in US and French SLE cohorts. **B.** Overall prevalence of IgE autoantibodies in SLE for seven tested autoantigens tested (dsDNA, Sm, SSA/Ro and SSB/La, APEX, MPG and CLIP4). **C.** Comparison of the levels of all tested autoreactive IgE antibodies (as in B) in US and French cohorts with disease activity as measured by SLEDAI score. Inactive, SLEDAI  = 0; Mild, SLEDAI >0 to <4; Active, SLEDAI ≥4. **D.** Prevalence of all tested autoreactive IgE’s in US and French SLE subjects with active disease. Mann Whitney test or Kruskal-Wallis with Dunn’s multiple comparisons test (more than two groups) was used to compare the different groups. Mean ± SEM is shown. **p<0.01; ***p<0.001, ****p<0.0001.

Roughly, 40–50% of SLE subjects tested did not have IgE autoantibodies to the four common SLE autoantigens (Figure 1B). To explore whether IgE antibodies to other autoantigens might be present in these subjects, we used Invitrogen’s ProtoArray to screen the sera of subjects from both the US and French cohort. As shown in Figure 4A, high levels of IgE autoantibodies to APEX nuclease 1 (APEX), N-methylpurine-DNA glycosylase (MPG), and CAP-GLY domain containing linker protein family member 4 (CLIP4) were found in some SLE subjects but were minimally detected in healthy controls. The difference in the relative amounts of these IgE autoantibodies between healthy controls and SLE subjects was significant. Minimal levels of IgG antibodies to these autoantigens were detected and no changes in their levels were observed in SLE subjects (Figure 4A). Inclusion of SLE subjects with these novel IgE reactivities with those having at least one of the four common SLE autoantibodies increased the prevalence of autoreactive IgE’s from approximately 57% (Figure 1B) to 65% (Figure 4B). Analysis of the association between these new IgE autoantibodies with disease activity revealed a highly significant association of APEX-, MPG-, and CLIP4–specific IgE’s with active disease (Figure 4C). Approximately 74% of SLE subjects who had active disease also had IgE’s that reacted to the four common SLE autoantigens (Figure S2A in File S1). The inclusion of IgE reactivity to at least one or more of the three novel autoantigens increased the proportion of active SLE subjects with these autoantibodies to almost 83% (Figure 4D). Thus, a large majority of SLE subjects with active disease have the presence of a circulating autoreactive IgE. Autoreactive IgE’s to APEX, MPG, and CLIP4 also showed a highly significant association with hypocomplementemia (Figure S3A in File S1) and with active nephritis (Figure S3B in File S1).

Of the 146 SLE subjects analyzed from the US and French cohort who were positive for autoreactive IgE’s to all seven autoantigens tested, 94 were positive for increased levels of autoreactive IgE whereas 107 were found to have increased levels of autoreactive IgG’s. Of this latter group, 76 subjects (71.1%) were positive for both (Figure S4A in File S1). Of the autoantigens tested, dsDNA-specific IgG and IgE and Sm-specific IgG and IgE showed a significant (p = 0.046) or highly significant (p<0.0001) correlation, respectively (Figure S4B in File S1).

## Discussion

It is well known that the presence of circulating autoantibodies of the IgA, IgM, and IgG isotypes are found in SLE. Moreover, the presence of circulating IgE autoantibodies has also been reported [10]. The presence of this diverse immunoglobulin response is not surprising given that SLE is characterized by abnormalities that involve hyperactive B and T cells (as well as other cell types like monocytes) resulting in polyclonal B-cell activation, increased numbers of plasma cells and autoantibody production. The mechanistic role of the different immunoglobulin isotypes in the onset and progression of SLE is, for the most part, still unclear. However, clues are emerging from studies in both mouse models and human disease. For example, IgA autoantibodies to phospholipids (anti-cardiolipin) have been shown to be a risk factor for thrombosis in SLE [18], [19] and in some SLE patients dsDNA-specific IgA is linked to active disease [20], [21], [22]. However, there are also reports that incidence of IgA-deficiency (characterized by the lack of IgA-producing B cells) is also higher in SLE and in such cases there appears to be no association to clinical parameters of disease [23], [24]. IgM autoantibodies (and in particular those to dsDNA) are also frequent in lupus. However, unlike their IgG counterpart, anti-dsDNA IgM appears to be inversely linked to the development of nephritis [25] and in mouse models there is evidence that such autoantibodies may have a protective role [26]. It has been proposed that the ratio of IgG/IgM anti-dsDNA antibodies might be a useful prognostic marker for lupus nephritis [27], although rigorous studies to test this hypothesis are yet to be conducted. The levels of IgG autoantibodies to dsDNA in serum are known to fluctuate with disease activity in SLE and they often increase prior to the flaring of disease activity. Because of their prevalence and specificity in SLE and their association with disease activity as well as with lupus nephritis, elevated levels of anti-dsDNA IgG is one accepted criterion in the diagnosis of SLE. In fact, anti-dsDNA of the IgG subclass is also frequently found in the kidney of SLE subjects with nephritis [28], [29] and some of these autoantibodies have been shown in various animal models to have pathogenicity [30], [31].

An understanding of the role of IgE autoantibodies in this disease and their link to clinical parameters of disease has been lacking. In the present work, we set out to rigorously explore the prevalence and the relevance of autoreactive IgE in SLE. Here, we find that autoreactive IgE to four common and three newly identified SLE autoantigens is present in 65% of SLE subjects as determined in both the US and French cohorts. This frequency is increased to almost 83% when one limits the cohort analysis to only SLE subjects with active disease, demonstrating that the prevalence of autoreactive IgE’s in SLE is not only common but that it is most often found in individuals with active disease. The findings clearly establish a link between autoreactive IgE and active nephritis, consistent with our previous preliminary results [12], [32], but also extend the previous findings by demonstrating that autoreactive IgE may be a reasonable predictor of SLE disease activity. Perhaps, the most unexpected finding of this study is that anti-dsDNA IgE can serve as a predictor of active disease and in combination with anti-dsDNA IgG enhances the predictive value. These findings suggest that measurement of the serum levels of autoreactive IgE’s (in combination with autoreactive IgG’s) may perhaps be a useful and more sensitive criterion in the diagnosis of SLE but further studies are clearly required.

An important question that remains to be addressed is the role of IgE in human SLE disease pathology. Although, some studies have demonstrated the presence of IgE in the kidney of SLE subjects [33] these reports are limited and the occurrence appears to be infrequent. This is consistent with mouse models of spontaneous lupus (such as the *lyn^−/−^* mice) where detection of IgE in the kidney has been difficult. One must also consider that the concentration of IgE in both the circulation and tissues is extremely low and that this immunoglobulin does not fix complement (a major participant in tissue damage in the lupus kidney), thus the pathological consequence of any IgE deposition in the kidney is unclear and the contribution of IgE to tissue damage would most likely occur through induction of proinflammatory responses. While the relationship between autoreactive IgE and autoantibody production in human disease is difficult to unravel, our current analysis on the combined cohorts tested for the common SLE autoantigens uncovered evidence of a significant correlation between increased levels of autoreactive IgE and increased levels of autoreactive IgG (Figure S4 in File S1). Since the absolute levels of autoreactive IgG’s or other immunoglobulins produced by a given SLE subject is highly variable (and influenced by the genetic makeup of the individual), evidence of a significant correlation in the levels of these two immunoglobulins was rather unexpected. This demonstrates that even in the presence of the multiple variables that can influence immunoglobulin responses in SLE, high levels of IgE are significantly correlated with the presence of high levels of IgG and vice versa, suggesting intimate co-regulation and perhaps supporting and/or complementary roles in disease progression.

## Conclusions

Increased IgE production has been documented in various autoimmune, inflammatory, and genetic diseases [34], [35], but beyond the role of IgE in allergic inflammation there is a lack of knowledge on the role of IgE in such diseases. The analysis conducted herein advances the relevance of IgE beyond allergies. It demonstrates that the incidence of autoreactive IgE in cohorts of both US and French SLE subjects is common and that IgE antibodies are generated to the common SLE autoantigens, as well as to autoantigens that appear to selectively elicit IgE and not IgG responses. The current analysis also demonstrates a significant relationship between the presence of high levels of autoreactive IgE, active disease and lupus nephritis. It suggests that autoreactive IgE (perhaps in combination with autoreactive IgG) may serve as a potential predictor of SLE. Replication of these findings in larger cohorts is essential. Moreover, longitudinal studies to assess the relationship of autoreactive IgE’s with disease onset or flares is necessary to ascertain its true relevance to disease activity and as potential predictors of disease onset. Additional studies are also required to determine the importance of autoreactive IgE’s, if any, in the development of lupus nephritis. Nonetheless, the findings clearly extend the relevance of IgE beyond allergies and demonstrate that autoantibody production of the IgE subclass is not rare in lupus, and perhaps is present in other autoimmunities.

## Supporting Information

File S1 **This file contains Figure S1–S4 and Table S1.** Figure S1, Combined analysis of US and French cohorts demonstrates similarities in the distribution of autoreactive IgE’s in the two cohorts. The forest plot represented, used a fixed-effect model of the mean relative levels of dsDNA, Sm, SSA/Ro and SSB/La IgE autoantibodies for the two distinct cohorts. SMD, standardized mean difference. Figure S2, High prevalence of IgE autoantibodies in SLE subjects with active disease. The prevalence of IgE autoantibodies to dsDNA, Sm, SSA/Ro and SSB/La in SLE subjects with active disease (SLEDAI ≥4) (A) or in SLE subjects with hypocomplementenemia (B, C3<80 mg/dl or C4<15 mg/dl) is shown. The percent of SLE subjects with positive dsDNA IgE levels was the reference point. Percent of subjects with Sm IgE had no dsDNA IgE, percent of subjects with SSA/Ro IgE had no dsDNA or Sm IgE, and the percent of subjects with SSB/La IgE had no detectable levels of dsDNA, Sm or SSA/Ro IgE. Figure S3, Association of novel autoreactive IgE levels (APEX, MPG and CLIP4) with nephritis (A) and complement levels (B). Low levels of complement were defined as C3<80 mg/dl or C4<15 mg/dl. Kruskal-Wallis with Dunn’s multiple comparisons test was used to compare the different groups. AU, Arbitrary Units. Mean ± SEM is shown. *p<0.05; **p<0.01; ***p<0.001; ****p<0.0001. Figure S4, Increased levels of dsDNA and Sm –specific IgE’s are associated with increased levels of their respective IgG counterparts. A. Analysis of the relationship between IgE and IgG in SLE subjects positive for all seven autoantigens (dsDNA, Sm, SSA/Ro, SSB/La, APEX, MPG, and CLIP4) tested in this study. Stacked bar (left panel) and analysis (table, right panel) of the combined number of French and US SLE subjects. Positivity was considered as a value over the mean +2SD of healthy controls for at least one of the aforementioned autoantigens. B. Spearman correlation between autoreactive IgE and IgG levels for dsDNA and Sm in the group of subjects with double positivity shown on a logarithmic scale. Table S1, Correlation of positive levels of autoreactive IgE with active nephritis. Analysis of the combined and individual US and French cohorts.
(DOCX)
