# Supplementary material for: Autoreactive IgE Is Prevalent in Systemic Lupus Erythematosus and Is Associated with Increased Disease Activity and Nephritis
Source: PLoS One. 2014 Feb 28;9(2):e90424. doi: 10.1371/journal.pone.0090424 (PMC3938730; doi:10.1371/journal.pone.0090424)
Supplement: File S1 — This file contains Figure S1–S4 and Table S1. Figure S1, Combined analysis of US and French cohorts demonstrates similarities in the distribution of autoreactive IgE’s in the two cohorts. The forest plot represented, used a fixed-effect model of the mean relative levels of dsDNA, Sm, SSA/Ro and SSB/La IgE autoantibodies for the two distinct cohorts. SMD, standardized mean difference. Figure S2, High prevalence of IgE autoantibodies in SLE subjects with active disease. The prevalence of IgE autoantibodies to dsDNA, Sm, SSA/Ro and SSB/La in SLE subjects with active disease (SLEDAI ≥4) (A) or in SLE subjects with hypocomplementenemia (B, C3<80 mg/dl or C4<15 mg/dl) is shown. The percent of SLE subjects with positive dsDNA IgE levels was the reference point. Percent of subjects with Sm IgE had no dsDNA IgE, percent of subjects with SSA/Ro IgE had no dsDNA or Sm IgE, and the percent of subjects with SSB/La IgE had no detectable levels of dsDNA, Sm or SSA/Ro IgE. Figure S3, Association of novel autoreactive IgE levels (APEX, MPG and CLIP4) with nephritis (A) and complement levels (B). Low levels of complement were defined as C3<80 mg/dl or C4<15 mg/dl. Kruskal-Wallis with Dunn’s multiple comparisons test was used to compare the different groups. AU, Arbitrary Units. Mean ± SEM is shown. *p<0.05; **p<0.01; ***p<0.001; ****p<0.0001. Figure S4, Increased levels of dsDNA and Sm –specific IgE’s are associated with increased levels of their respective IgG counterparts. A. Analysis of the relationship between IgE and IgG in SLE subjects positive for all seven autoantigens (dsDNA, Sm, SSA/Ro, SSB/La, APEX, MPG, and CLIP4) tested in this study. Stacked bar (left panel) and analysis (table, right panel) of the combined number of French and US SLE subjects. Positivity was considered as a value over the mean +2SD of healthy controls for at least one of the aforementioned autoantigens. B. Spearman correlation between autoreactive IgE and IgG levels for dsDNA and Sm in the group of sub [file pone.0090424.s001.docx]

**Figure S1.**

**
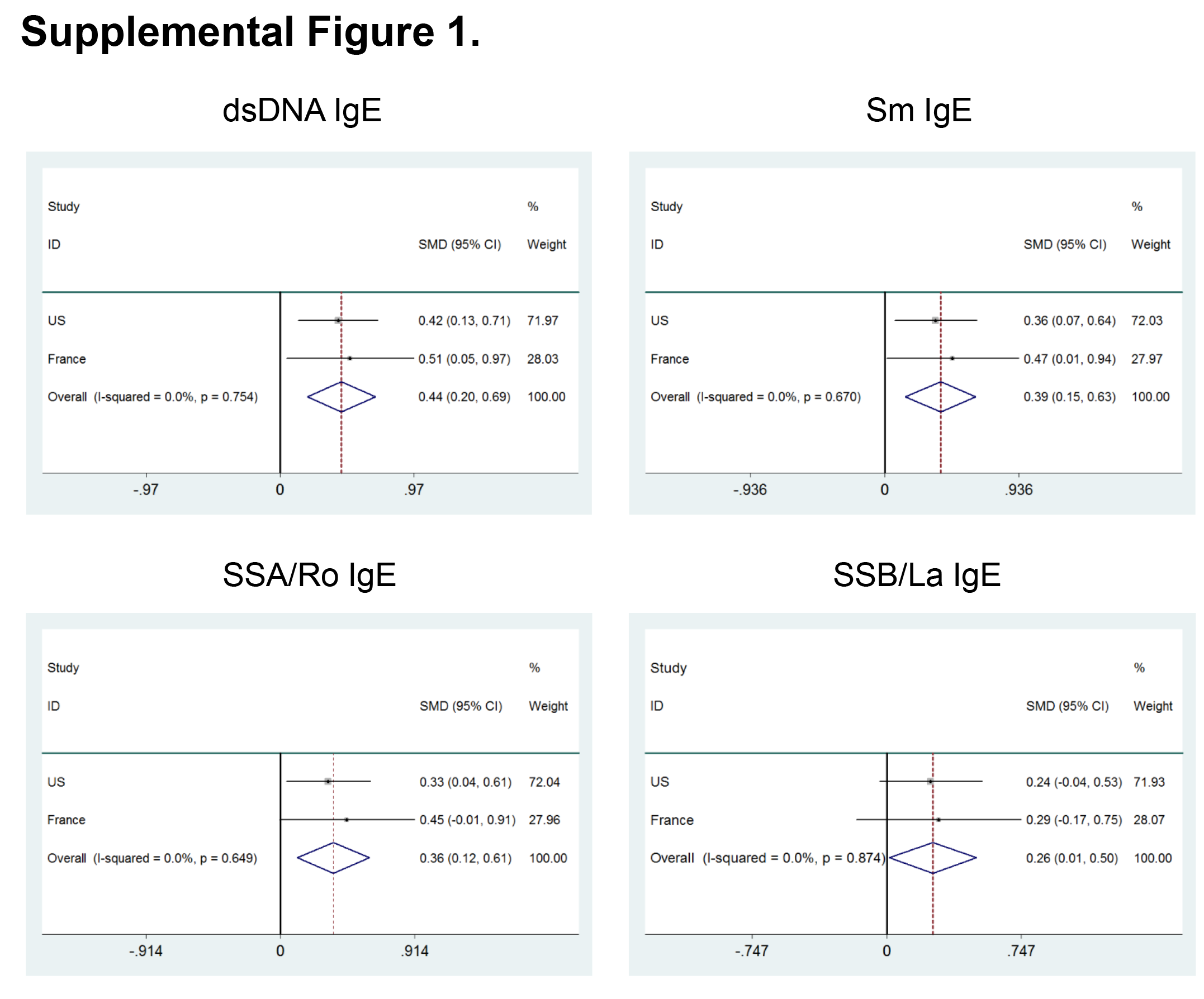
**

**Figure S2.**


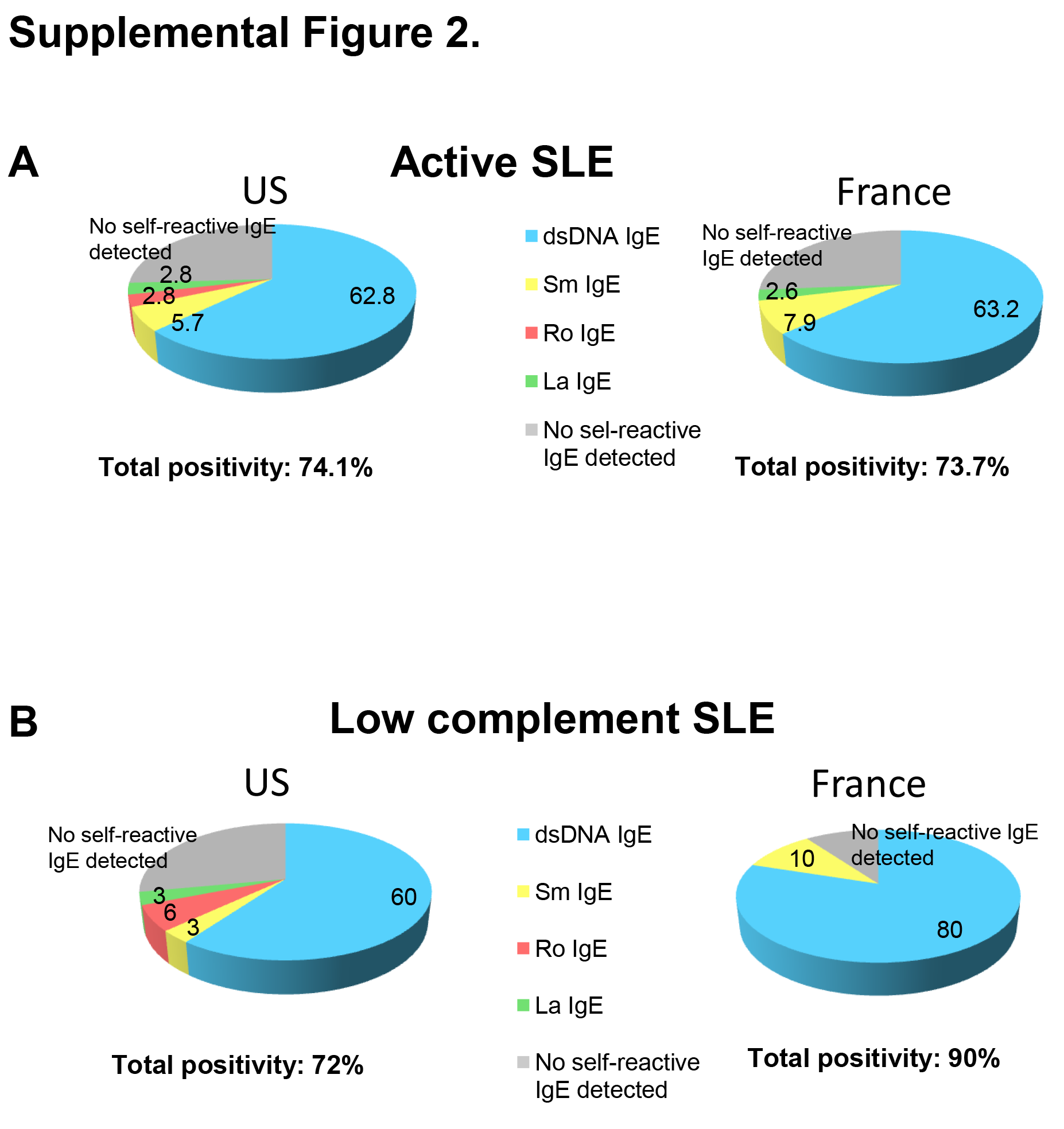


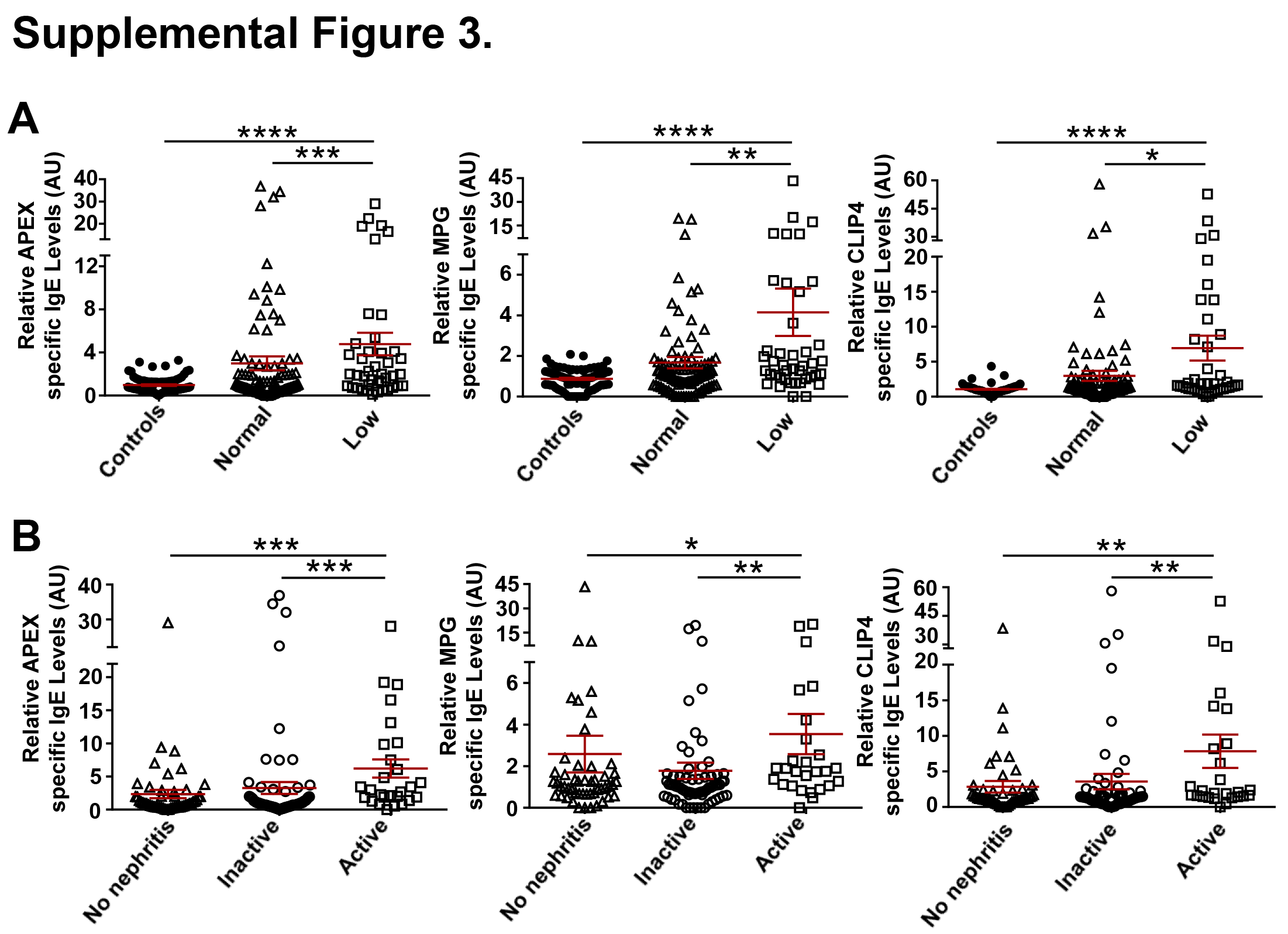
**Figure S3.**


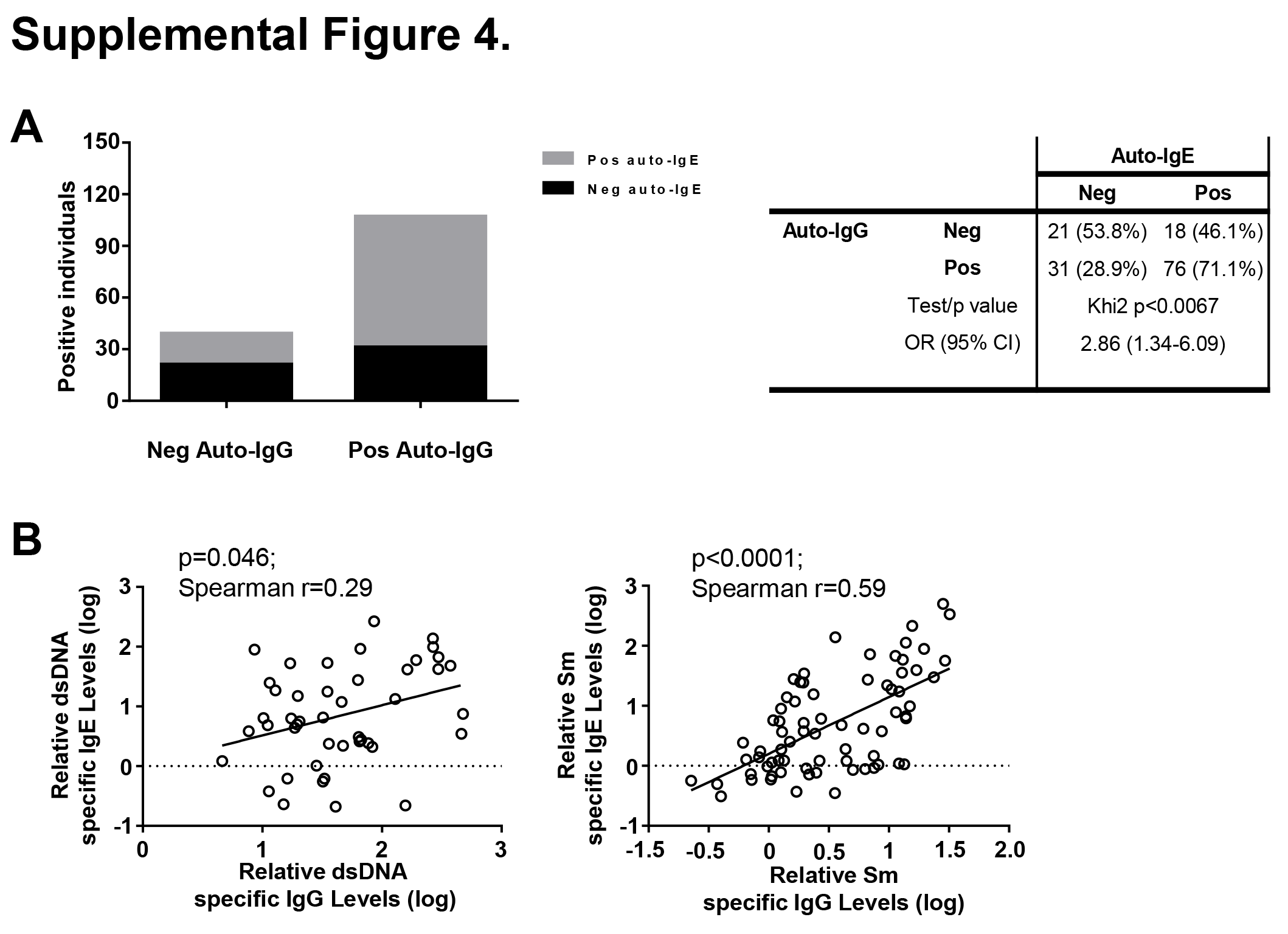
**Figure S4.**

**Table S1.**

|  |  | **Anti-dsDNA IgE** | | **Anti-Sm IgE** | | | **Anti-Ro/SSA IgE** | | | | **Anti-La/SSB IgE** | |
| --- | --- | --- | --- | --- | --- | --- | --- | --- | --- | --- | --- | --- |
|  |  | **Neg** | **Pos** | **Neg** | | **Pos** | **Neg** | **Pos** | | **Neg** | | **Pos** |
| Active nephritis Combined | **No** | 104 | 49 | 114 | 38 | | 114 | | 38 | | 123 | 29 |
|  |  | (68.0%) | (32.0%) | (75.0%) | (25.0%) | | (75.0%) | | (25.0%) | | (80.9%) | (19.1%) |
|  | **Yes** | 18 | 25 | 22 | 18 | | 23 | | 17 | | 29 | 11 |
|  |  | (41.9%) | (58.1%) | (55%) | (45.0%) | | (52.5%) | | (42.5%) | | (72.5%) | (27.5%) |
|  | **p value** | p=0.0018 | | p=0.0133 | | | p=0.029 | | | | p=0.243 | |
|  | **OR (95% CI)** | 2.95 (1.47-5.90) | | 2.45 (1.19-5.06) | | | 2.22 (1.07-4.59) | | | | 1.609 (0.72-3.59) | |
| Active nephritis US | **No** | 71 | 33 | 74 | 30 | | 77 | | 27 | | 82 | 22 |
|  |  | (68.3%) | (31.7%) | (71.2%) | (28.8%) | | (74.0%) | | (26.0%) | | (78.8%) | (21.2%) |
|  | **Yes** | 6 | 7 | 6 | 7 | | 7 | | 6 | | 7 | 6 |
|  |  | (46.2%) | (53.8%) | (46.2%) | (53.8%) | | (53.8%) | | (46.2%) | | (53.8%) | (46.2%) |
|  | **p value** | p=0.20 | | p=0.13 | | | p=0.23 | | | | p=0.09 | |
|  | **OR (95% CI)** | 2.51 (0.78-8.57) | | 2.87 (0.89-9.275) | | | 2.44 (0.75-7.92) | | | | 3.19 (0.97-10.48) | |
| Active nephritis France | **No** | 33 | 16 | 40 | 8 | | 37 | | 11 | | 41 | 7 |
|  |  | (67.3%) | (32.7%) | (83.3%) | (16.7%) | | (77.1%) | | (22.9%) | | (85.4%) | (14.6%) |
|  | **Yes** | 12 | 18 | 16 | 11 | | 16 | | 11 | | 22 | 5 |
|  |  | (40.0%) | (60.0%) | (59.3%) | (40.7%) | | (59.3%) | | (40.7%) | | (81.5%) | (18.5%) |
|  | **p value** | p=0.017 | | p=0.021 | | | p=0.104 | | | | p=0.90 | |
|  | **OR (95% CI)** | 3.094 (1.20-7.95) | | 3.44 (1.17-10.12) | | | 2.31 (0.84-6.42) | | | | 1.33 (0.38-4.69) | |

OR, Odds Ratio

CI, Confidence Interval
